# Expanding Pharmacy Capacity for Patient-Centered Reproductive Health Services

**DOI:** 10.3390/pharmacy8040236

**Published:** 2020-12-09

**Authors:** Anna Pfaff, Sally Rafie

**Affiliations:** 1 Provide Inc., Round Rock, TX 78683, USA; 2 Birth Control Pharmacist, San Diego, CA 92122, USA; sally@birthcontrolpharmacist.com

**Keywords:** contraception, emergency contraception, abortion, pharmacy, pharmacist, patient-centered care, family planning, referrals

## Abstract

In the United States, patients face increasing practical barriers and concerns about stigma when seeking sexual and reproductive healthcare, specifically family planning services involving hormonal contraception, emergency contraception, and abortion. The pharmacist is a member of the interprofessional care team with the ability to provide non-judgmental, high-quality, and patient-centered care. The community pharmacy setting itself offers specific advantages which promote access, including availability in most neighborhoods, broad hours of operation, lack of need for an appointment, and a stigma-free space which is frequented for other goods and services. This commentary suggests specific ways for pharmacies to improve access to contraception, emergency contraception, and abortion in line with national quality recommendations. Particular focus is given to the intersection of sexual and reproductive health resources and referrals within the pharmacy profession as well as the training and technical assistance tools which can help address unmet patient need.

## 1. Current Landscape of Reproductive Health Access

Sexual and reproductive health services are essential to the overall wellbeing of individuals, families, and communities. An estimated 99% of sexually active female-bodied people have used contraception; 20% report using emergency contraception; approximately 25% will obtain an abortion in their lifetime [1,2]. All of these choices allow individuals to exercise control of their desired family size and birth spacing, and in this context, can be understood as components of comprehensive “family planning”. In the United States, individuals face noteworthy barriers to equitable access to these services.

Consider the reality of a person in need of reliable contraception. They may lack consistent transportation, face childcare difficulties, have limited insurance coverage, and a work schedule which prohibits them from easily attending medical appointments. Living in a rural area is associated with a number of healthcare disparities and today an estimated 19 million female-bodied people in need of publicly funded contraception services live in “contraception deserts”, defined as areas which lack reasonable access to a health center that provides a full range of birth control methods [3]. If birth control access is delayed or disrupted, these challenges must be navigated in an accelerated timeline to obtain emergency contraception. Finally, the accumulation of these barriers, as well as legal setbacks and fear of stigma, must be addressed if this person experiences an unintended pregnancy and seeks an abortion. Streamlining access to all these services is the best practice to reduce delays in care and prevent subsequent undesired health outcomes. 

Contraception and abortion have historically been difficult to access due to overt legal restrictions and covert influences, such as societal stigma. Trends illustrate that, in the United States, patients face increasing barriers to these services. Geographic distribution of healthcare services is of primary concern. Currently, 89% of US counties are without an abortion provider which leaves this service effectively inaccessible for many people, particularly those who are poor or who live long distances from the nearest provider [2]. Additionally, the number of facilities able to provide a comprehensive array of contraceptive services is shrinking. The Title X Family Planning Program is a federal grant program for low-income patients to receive family planning and reproductive health services. In 2019, an overhaul of Title X regulations, collectively known as the “domestic gag rule”, prohibited sites receiving these funds from referring for abortion, eliminated requirements to provide non-directive pregnancy options counseling, and encouraged participation in the program by organizations which only offer limited family planning methods [4]. It is estimated that approximately one-quarter of Title X sites left the federally funded program because of these changes and, as a result, halved the domestic family planning network’s capacity, jeopardizing care for over 1.6 million patients [5]. This shift is particularly relevant in light of current events in 2020. A survey from the National Family Planning and Reproductive Health Association (NFPRHA) found that the majority of US adults (52%) feel it is more essential for individuals to have access to birth control during a pandemic [6].

Funding and insurance coverage present another practical barrier. States that have not expanded Medicaid impose roadblocks to contraception for individuals who are not eligible for coverage, either through state funded programs or other avenues [7]. Although federal law requires health insurance coverage for the full range of contraceptive methods, refusal clauses from religiously affiliated employers and insurers are permitted [8]. Federal Medicaid funds are prohibited from covering abortion services by the Hyde Amendment, and many states have additional restrictions on abortion including waiting periods, abortion facility requirements, and gestational age limits [9]. These barriers translate into clinic closures and increased practical costs for patients including loss of time at work, additional childcare coverage, and the potential for multiple trips outside of one’s county or state for care [10]. 

Across the country, stigma plays a significant role in limiting access. Misinformation and cultural bias surrounding reproductive health, including that from healthcare providers, contribute to this. In times of crises and natural disasters, including coronavirus disease 2019 (COVID-19), individuals face further structural shortcomings such as disruption in supply chains, travel restrictions, prioritization of emergent medical visits, omission of reproductive health services from recognized “essential services”, and state legislation which threatens temporary closures of abortion care facilities [11].

Although barriers to reproductive health services are complex and far-reaching, pharmacists have the power to reduce difficulties for patients by providing quality services, accurate information, and seamless connections to other care providers. This commentary suggests specific ways that pharmacies can meet patient needs for contraception, emergency contraception, and abortion through referrals, training, and technical assistance tools. 

## 2. Quality Family Planning Services and Referrals

The 2014 federal report Providing Quality Family Planning Services: Recommendations of CDC and the US Office of Population Affairs (QFP) uses the Institute of Medicine definition for quality healthcare as having the following attributes: safety, effectiveness, timeliness, efficiency, accessibility, equity, value, and a client- or patient-centered approach [12]. This standard is consistent with committee opinions from the American College of Obstetricians and Gynecologists and the American Academy of Pediatrics [13,14]. 

In practice, these recommendations outline interactions which inform patients of all options available to them and which are respectful of, and responsive to, individual patient preferences, needs, and values. Research in health and human service fields shows that supportive and effective recommendations from a provider can help prevent delays in accessing care and the subsequent health outcomes [15]. 

## 3. The Role of Pharmacists in Family Planning Access

Pharmacies are a trusted source of over-the-counter family planning supplies, including pregnancy tests, condoms, and emergency contraception. Pharmacists also provide patient care in collaboration with other healthcare providers by dispensing prescriptions for an array of reproductive health uses, including prescription hormonal contraception, ulipristal acetate for emergency contraception, and misoprostol, which has multiple indications in obstetric and abortion care, among other reproductive health medications. Pharmacists in twelve states are currently permitted by statewide protocol, standing order, or collaborative practice agreement to prescribe and dispense hormonal contraception directly to patients in the pharmacy setting [16]. An additional four states and the District of Columbia are in the process of similarly expanding access [16]. These professional responsibilities present opportunities to optimize patient care in a timely and accessible manner. 

The American Pharmacists Association’s code of ethics outlines a professional commitment to respect the patient/pharmacist relationship; to uphold the autonomy and dignity of each patient; to extend caring, compassionate, and confidential services [17]. Similar to medical providers, pharmacists who prescribe, dispense, and/or educate on reproductive health should do so in line with these professional expectations. 

Pharmacy teams can improve coordination of care, increase patient satisfaction, remove practical barriers, and adhere to professional expectations through the integration of high-quality, patient-centered resources and referrals. This requires a willingness to provide services free of personal bias, share knowledge of up to date resources, complete training on patient-centered approaches, and integrate technical assistance tools into practice. A review of extant online resources identified no current continuing education programs designed for pharmacists addressing high-quality resources and referrals for reproductive health services in this country. Additionally, the technical assistance tools to complement these skills are absent from workforce development and standardized pharmacy curricula in the United States. 

## 4. Opportunities to Improve Quality Family Planning Services at the Pharmacy

In addition to applying relevant recommendations outlined in QFP, there are additional considerations to optimize pharmacist practices and community pharmacy settings with regard to family planning services. Studies have evaluated pharmacies and pharmacists for family planning services—elements ranging from product availability and accessibility, youth-friendly spaces and services, accuracy of information, to availability of clinical services [18,19,20,21,22,23]. While these studies have found some encouraging results, they have also identified many opportunities for improvement. 

Based on clinical pharmacy experience, peer-reviewed research, and industry-recognized best practices in patient care, the following recommendations for improved family planning counseling and referrals at the pharmacy were compiled. Though this list is far from comprehensive, it can be used by pharmacists and other pharmacy stakeholders when designing pharmacy spaces, creating new programs, or providing clinical services. 

### 4.1. Safety 

#### Pharmacists Follow National Guidelines When Providing Contraception Services

Pharmacists should adhere to the US Centers for Disease Control and Prevention’s guidelines related to contraception, the US Medical Eligibility Criteria (US MEC) for Contraceptive Use [24], and the US Selected Practice Recommendations (US SPR) for Contraceptive Use [25] guidelines to ensure patient safety. 

### 4.2. Effectiveness 

#### 4.2.1. Pharmacists Practice to the Top of Their Licenses

Pharmacists should prescribe, dispense, and administer a full range of Food and Drug Administration (FDA)-approved contraceptives, to the extent allowed by state law. Pharmacist scope of practice has been expanded to include direct contraceptive care in selected states across the country. Increasing pharmacy capacity to include these skills requires specific training on method effectiveness including consideration of patient situations, and potential harms and benefits.

#### 4.2.2. Pharmacists Provide Accurate Information on Reproductive Health Products and Services

Integrating medically comprehensive, health literate patient educational material will support pharmacy staff to dispel myths about contraception, emergency contraception, and abortion. In particular, pharmacy staff should provide accurate information about emergency contraception given the acute nature of its use, which requires staff to stay abreast of updates to product labeling and clinical guidelines. 

### 4.3. Patient-Centered Approach 

#### 4.3.1. Confidential Space Is Secured and Utilized in the Pharmacy

Creating private locations for patient consultation creates a safe environment, increases patient trust, and decreases perceived stigma. This may include a dedicated office within the pharmacy, partitioned cubicle space, and/or a white noise machine. 

#### 4.3.2. Comprehensive Counseling Is Provided

Making a quality referral means the provider plays an active role in decision-making but refrains from imposing their personal beliefs [26]. Prepare staff to provide client-led counseling, including education on methods not available at the pharmacy, particularly long-acting reversible contraceptives (i.e., intrauterine device, subdermal implant). 

#### 4.3.3. Pharmacy Policies Require Patient-Centered Information and Referrals

Expectations of high-quality referrals to improve patient satisfaction should be reflected by leadership in policies and procedures for pharmacy professionals. State pharmacy boards and associations can adopt and encourage the use of templates to support integration into practice. 

### 4.4. Timeliness

#### 4.4.1. Pharmacies Have the Full Range of Over-the-Counter and Prescription-Only Reproductive Health Products and Medications Available

Due to the acute nature of the need, these products and services should be provided to patients in a timely manner. Displaying emergency contraception, pregnancy tests, internal and external condoms in front of the counter reduces obstacles to prompt uptake. All emergency contraceptive pill products should be kept in stock. Other routine contraceptive medications should be kept in stock or available for order. 

#### 4.4.2. Pharmacists Integrate Local and Nationwide Referral Resources

Searchable databases curated by recognized health and research experts can streamline the collaboration between the pharmacy and other health professionals, even when resources are located outside of county or state lines. 

### 4.5. Efficiency 

#### 4.5.1. Pharmacies Obtain Recognition for Reproductive Health Capacity

There is an opportunity for national and state organizations to create programs to recognize and award pharmacies which meet best practice standards. Visual displays of pharmacies which offer effective, high-quality family planning services can increase the value of this local healthcare resource for patients in need of appropriate services, supplies, or referrals. 

#### 4.5.2. Training and Technical Assistance Resources Are Integrated into Pharmacy Education and Staff Onboarding

Best practice strategies for developing pharmacy skills and processes can be easily added to the core academic curriculum. Community pharmacies can include these resources in orientation materials, core competencies, and staff training.

### 4.6. Accessibility

#### 4.6.1. Environmental Prompts Are Displayed

Posting signage which advertises product availability and staff consultation can increase public awareness and patient uptake. Displaying information about reproductive health services and referrals on patient-facing webpages, mobile apps, and virtual communications increases opportunities for access. 

#### 4.6.2. Reproductive Health Products Are Included in Online Ordering, Delivery, and Telehealth Services

Including contraception and emergency contraception in telehealth and same-day delivery services can reduce transportation and scheduling restrictions. Online services are especially valuable to populations with higher use of internet-based commerce, and patients with restricted mobility due to health or social conditions, as well as during times of natural disaster. 

### 4.7. Equity

#### 4.7.1. Pharmacy Staff Have the Skill and Willingness to Dispense Reproductive Health Products, Regardless of Their Personal Feelings

Prompts and phone scripts to guide staff in patient-centered referral which uphold autonomy, dignity, and honesty support the best outcomes for patients. Awareness of personal bias, destigmatizing language and behaviors, and referral best practices can reduce barriers to care. 

#### 4.7.2. Staff Are Proactively Screened for Objections to Providing Information and Referral

Interview questions and scenarios involving reproductive health can be utilized by hiring personnel to screen for potential conflicts to the provision of comprehensive patient care. Internal systems of communication and triage are developed within the pharmacy team to ensure timely patient care. 

#### 4.7.3. Pharmacies Adapt Services for Various Patient Populations

Pharmacy services should be adapted to meet the needs of various populations, including Black, Indigenous, People of Color (BIPOC), youth, patients who identify as lesbian, gay, bisexual, transgender, queer, intersex, asexual, and/or two spirit (LGBTQIA2+), and differently abled patients. While use of language services is encouraged, pharmacies should consider what additional adaptations can be made to their services and communications to reach all patients in their community. 

### 4.8. Value 

#### 4.8.1. Resources for Cost and Coverage Are Explored with Patients

Ensure that services are cost-effective by highlighting generic alternatives, funding programs, insurance opportunities, and similar cost-reducing options for patients seeking family planning services. 

#### 4.8.2. Payment for Services and Reimbursement for Medications Are Prioritized

Existing payment pathways should be utilized. 

## 5. Implementation Considerations

Training and technical assistance tools which support the integration of patient-centered family planning services at the pharmacy are now available across the country. Meeting Reproductive Health Needs at the Pharmacy, https://birthcontrolpharmacist.com/referrals, is an asynchronous, standalone, home study, and continuing pharmacy education program, co-created by Birth Control Pharmacist, an expert in pharmacy training and advocacy, and Provide Inc., a non-profit specializing in stigma reduction and referrals for unintended pregnancy. The curriculum’s objectives are to address learning gaps around reproductive health barriers; provide resources for local referrals; apply best practices in the provision of referrals for contraception, emergency contraception, and abortion. Technical assistance materials, including downloadable communication guides, patient education material, checklists, and links to nationwide resources supplement the learning tool. 

An initial cohort of self-selecting participants (*n* = 125) filled registration capacity for the online launch of this program in less than one week, illustrating interest in the topic. Prior to the training, participants used a Likert scale to indicate their baseline willingness to help a patient access family planning services. Responses ranged from very unwilling, unwilling, willing, to very willing, and were coded from 0 to 4. This self-assessment was again administered following the training to capture change in attitude. Results (pre *n* = 74, post *n* = 44) showed increases in willingness to help a patient access contraception (average score of 3.3 to 3.8, a 15% increase), emergency contraception (average score of 3.2 to 3.7, a 15% increase), and abortion (average score of 2.5–3.1, a 24% increase). Participants were also asked about changes in self-reported skill (ranging from very unskilled to very skilled) helping a patient access these same services. Results showed increases in perceived skill helping a patient access contraception (average score of 2.6 to 3.7, a 53% increase), emergency contraception (average score of 2.8 to 3.5, a 25% increase), and abortion (average score of 1.2 to 2.7, an 80% increase).

## 6. Conclusions

Professional ethical expectations indicate that pharmacists play a key role in building a healthcare system where everyone is treated with dignity and respect [17]. Addressing the current landscape of disparities requires that all healthcare workers have the tools and support to offer high-quality patient care. Equipping community pharmacies with specialized skills in family planning needs will increase their ability to meet patient and community needs, and subsequently reduce barriers to access. Newly developed learning resources, available to pharmacists and support staff, are a bridge to successfully implementing practices which reduce stigma, increase patient satisfaction, and improve health outcomes. Opportunities exist for further research, implementation, and evaluation of family planning resources and referrals in the pharmacy setting.
